# ACTH Regulation of Adrenal SR-B1

**DOI:** 10.3389/fendo.2016.00042

**Published:** 2016-05-13

**Authors:** Wen-Jun Shen, Salman Azhar, Fredric B. Kraemer

**Affiliations:** ^1^The Division of Endocrinology, Stanford University, Stanford, CA, USA; ^2^Geriatric Research, Education and Clinical Center, Veterans Affairs Palo Alto Health Care System, Palo Alto, CA, USA

**Keywords:** adrenal, cholesterol, ACTH, SR-B1

## Abstract

The adrenal gland is one of the prominent sites for steroid hormone synthesis. Lipoprotein-derived cholesterol esters (CEs) delivered *via* SR-B1 constitute the dominant source of cholesterol for steroidogenesis, particularly in rodents. Adrenocorticotropic hormone (ACTH) stimulates steroidogenesis through downstream actions on multiple components involved in steroidogenesis. Both acute and chronic ACTH treatments can modulate SR-B1 function, including its transcription, posttranscriptional stability, phosphorylation and dimerization status, as well as the interaction with other protein partners, all of which result in changes in the ability of SR-B1 to mediate HDL-CE uptake and the supply of cholesterol for conversion to steroids. Here, we provide a review of the recent findings on the regulation of adrenal SR-B1 function by ACTH.

The adrenal gland, in addition to the gonads, is one of the prominent sites where steroid hormones are synthesized (1–5). Cholesterol is the common precursor for steroidogenesis, which involves the contribution from multiple enzymes and requires the conversion of cholesterol to pregnenolone as the initial step of a multistep process. Pregnenolone is subsequently metabolized to produce various biologically active steroids in a tissue-specific manner. In general, this process is thought to involve five major steps: (1) cholesterol acquisition through *de novo* synthesis and/or uptake from lipoproteins and stored as cholesterol esters (CEs) in lipid droplets (LDs), (2) cholesterol mobilization from CEs that are stored in LDs, (3) trafficking of cholesterol to the cytochrome P450 side-chain cleavage enzyme (P450scc, CYP11A1) at the inner mitochondrial membrane (IMM), following cholesterol trafficking to the outer mitochondrial membrane (OMM), (4) production of pregnenolone by CYP11A1 through cleavage of the cholesterol side-chain, and (5) efflux of pregnenolone from the mitochondria to the endoplasmic reticulum (ER), where enzymes convert it into intermediates that shuttle between mitochondria and ER to produce progestins, estrogens, androgens, glucocorticoids, or mineralocorticoids in a tissue-specific manner (2, 5).

The adrenal gland is a compound endocrine gland composed of two developmentally unrelated tissues, an outer layer of adrenal cortex and an inner layer of adrenal medulla. The adrenal cortex is the site of steroid hormone synthesis and produces three classes of steroid hormones. These are glucocorticoids (cortisol and corticosterone), mineralocorticoids (aldosterone), and androgens [androstenedione and dehydroepiandrosterone (DHEA)]. The cells of the adrenal zona glomerulosa, which is the outermost layer of the adrenal cortex, synthesize aldosterone in response to angiotensin II (1, 2), whereas the cells of the adrenal cortical zona fasciculata–reticularis produce cortisol, corticosterone, or androgens [androstenedione and DHEA/dehydroepiandrostenedione sulfate (DHEAS)] in response to adrenocorticotropic hormone (ACTH) stimulation. Humans synthesize cortisol, but because CYP17 is poorly expressed in the zona fasciculata in rats and mice, consequently, corticosterone is the dominant glucocorticoid produced in rodents. The adrenal steroidogenic pathways are illustrated in Figure 1.

**Figure 1:**
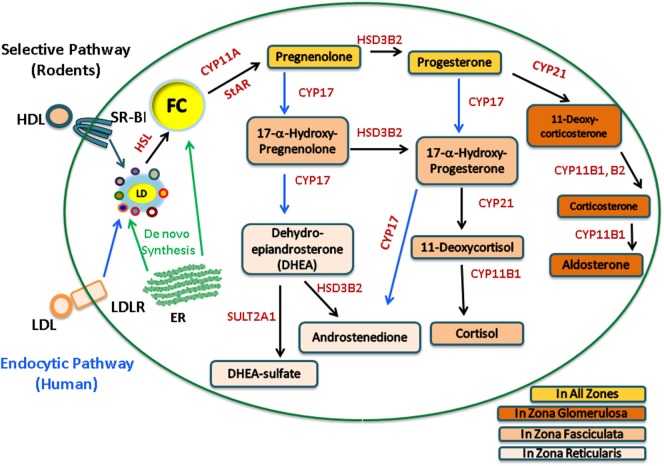
**Human and rodent adrenal steroidogenic pathways**. In the adrenal, upon stimulation, cholesterol esters (CE) from the LDL endocytic pathway (human) or the SR-B1 selective pathway (rodents) are hydrolyzed by hormone-sensitive lipase (HSL) to release free cholesterol (FC) as the common precursor for steroidogenesis. FC, which can also be synthesized *de novo* within the ER, traffics to the outer mitochondrial membrane and is then transported into the inner mitochondrial membrane by StAR, where it is cleaved by CYP11A to pregnenolone, a common precursor for all other steroid hormones. The adrenal cortex is the site of steroid hormone synthesis and different steroidogenic enzymes are expressed in cells located within different zones of the adrenal cortex, resulting in different classes of steroid hormones being released from different layers. The cells of the zona glomerulosa synthesize aldosterone, the cells of the zona fasciculata synthesize cortisol, and the cells of the zona reticularis produce androgens. In rodents, there is a very low level of CYP17; consequently, corticosterone is the dominant glucocorticoid produced.

All the steroid hormones synthesized within the adrenal cortex utilize cholesterol as the common precursor. For cells that produce polypeptide hormones, large amounts of mature hormones can be stored ready for rapid release; however, there is very little steroid hormone storage in steroidogenic cells. Therefore, upon stimulation, there is a rapid response from the steroidogenic cells to synthesize new steroids (3, 4), and with this a requirement for a constant supply of the precursor cholesterol to be converted to steroid hormones. The precursor cholesterol for steroidogenesis can be derived from a combination of sources (5–7): (1) *de novo* cellular cholesterol synthesis, (2) the mobilization of CEs stored in LDs, and (3) lipoprotein-derived CEs delivered through endocytic uptake, which is mediated by the LDL receptor or “selective” cellular uptake *via* the scavenger receptor, class B type 1 (SR-B1).

SR-B1 has been shown to be a HDL receptor and can mediate selective uptake of lipoprotein (HDL)-derived CEs both *in vitro* and *in vivo* (8–11). In the selective CE uptake pathway that is mediated by SR-B1, CE-rich lipoproteins bind on the cell surface and deliver the CEs from the hydrophobic core of the lipoproteins to the inside of the cells. The lipoprotein particles remain intact at the cell surface and can be further recycled to deliver more CEs to the cells (12). In contrast, CEs delivered *via* LDL receptor-mediated lipoprotein uptake are hydrolyzed by lysosomal acid lipase, releasing unesterified free cholesterol (FC) from lysosomes that traffics to the ER and plasma membrane (PM) and is then available to traffic to mitochondria (13, 14). CEs delivered *via* SR-B1 appear to be incorporated directly into LDs (15, 16) and must be hydrolyzed to FC before being used in steroidogenesis. Upon ACTH treatment, adrenal CE stores within LDs are rapidly depleted (17) through the action of hormone-sensitive lipase (HSL), the major neutral cholesteryl ester hydrolase expressed in the adrenal gland (18). This newly released FC from stored LDs is the preferred source of cholesterol. Following LD depletion, lipoprotein-derived CEs delivered *via* SR-B1 become the dominant source of cholesterol for steroidogenesis in rodents (19–21).

Adrenal fasciculata–reticularis cell steroidogenesis is under the regulation of tropic hormone ACTH and is subject to both acute (14, 22–25) and chronic regulation (14, 26–29). ACTH binds to its G protein-coupled receptors, leading to the activation of adenylate cyclase, which generates cAMP and activates cAMP-dependent protein kinase (PKA) (30–33). Stimulation of the cAMP–PKA signaling cascade exerts both acute and chronic effects on the regulation of steroid hormone production (Figure 2). On the other hand, angiotensin (AII) stimulates aldosterone biosynthesis in adrenal glomerulosa cells, and its actions are primarily mediated by the protein kinase C signaling cascade, whereas potassium can also stimulate aldosterone production through Ca^2+^-calmodulin-dependent kinase (34). Both acute and chronic ACTH treatments can modulate SR-B1, including its expression levels as well as its phosphorylation status, dimerization, and the interaction with other protein partners, all of which result in changes of SR-B1 function. Here, we aim at providing a review of the most recent findings relevant to these aspects.

**Figure 2:**
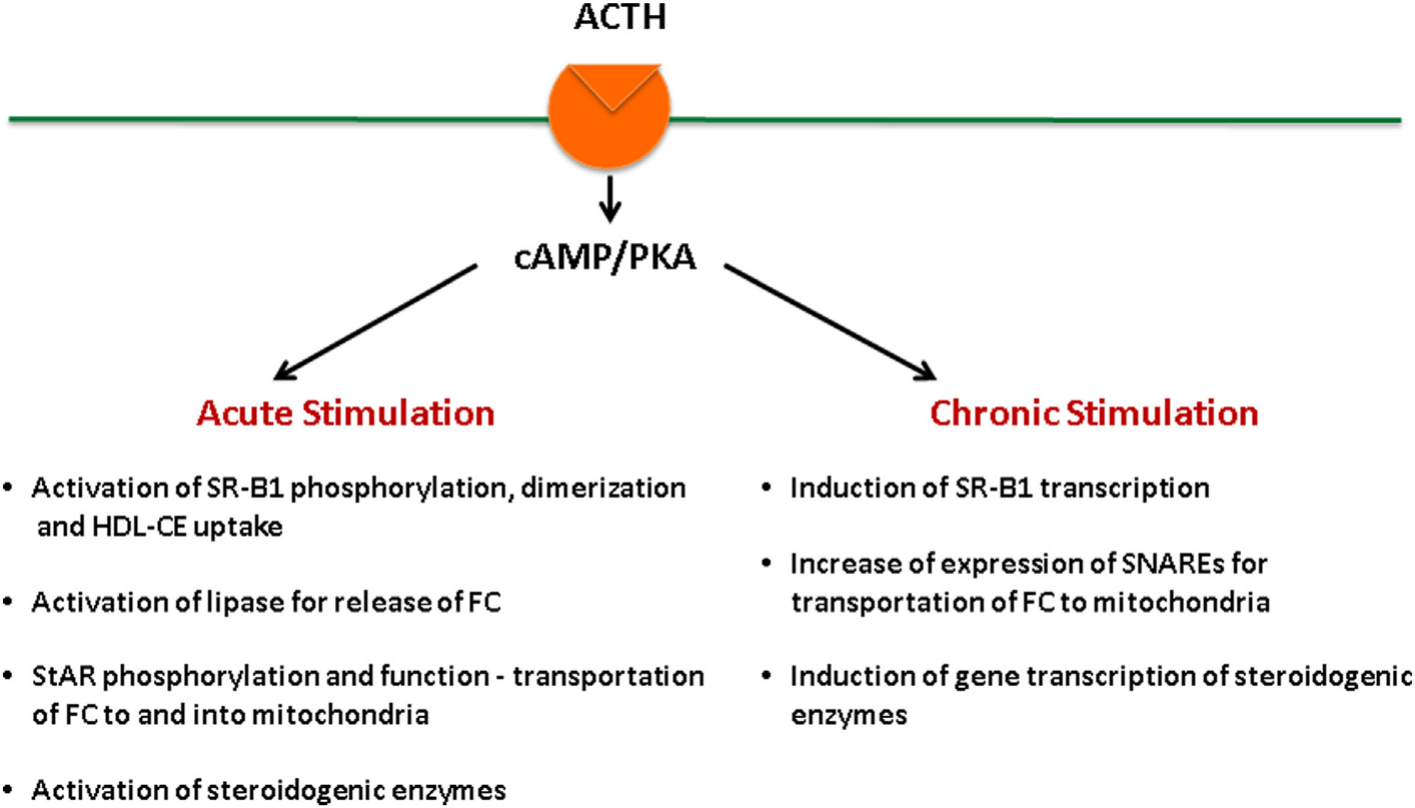
**ACTH regulation of steroidogenesis in the adrenal**.

## ACTH Stimulation Regulates the Expression of SR-B1

Initial studies established a functional correlation between SR-B1 expression, HDL-CE uptake, and the ability of steroidogenic cells to produce steroid hormones (8, 35–40). Also, in both lower vertebrates (i.e., turtle) and the fruit fly, there is an increase in SR-B1 expression that correlates with the peak cholesterol flux required during their developmental stages (41). Follow-up studies revealed that the bulk of the cholesterol for steroidogenesis is provided from the selective uptake pathway, which is mediated by SR-B1.

Both rodent and human adrenals express exceptionally high levels of SR-B1; indeed, the highest expression level of SR-B1 per gram of tissue has been reported for rodent adrenals (42, 43). *In vivo* treatment of rats and mice and *in vitro* treatment of cultured rodent adrenocortical cells with ACTH increased SR-B1 expression both at the mRNA level and that of SR-B1 protein (8, 44). In one of our recent studies, the level of SR-B1 protein in rat adrenals shows a trend for increased SR-B1 expression as early as 1 h after treatment with ACTH (45). Similar stimulation of SR-B1 expression and regulation by ACTH was also demonstrated for cultured human adrenal cells (8, 43, 46, 47). Indeed, low levels of SR-B1 mRNA expression seen in normal adrenal tissue adjacent to adenomas causing Cushing’s syndrome, where plasma ACTH levels are reduced, is consistent with the notion that the regulation of human SR-B1 is possibly similar to that reported for rodents (43).

Much of the regulation of SR-B1 expression is at the level of transcriptional control. The promoter region of both human and rat SR-B1 contains binding sites for steroidogenic factor-1 (SF-1) (48), which is one of the major transcription factors involved in cAMP regulation of the *SR-B1* gene (49). In addition, the rat promoter has two sterol-responsive elements (SREs) that can bind sterol-responsive element-binding protein (SREBP)-1a and regulate *SR-B1* gene expression in response to altered intracellular sterol levels (50).

Further studies demonstrated that the promoter region of SR-B1 contains sites that can bind both positive and negative regulators. For the expression of SR-B1 in liver and adipose tissue, transcription factors, such as the liver X receptors α and β (LXRα and LXRβ) and the peroxisome proliferator-activated receptor α (PPARα), have been shown to positively regulate the expression of the human *SR-B1* gene in response to oxysterols and fibrates, respectively (51, 52). Other positive regulators include the liver receptor homolog 1 (LRH-1) (53) as well as the estrogen receptors α and β (ERα and ERβ), which bind to three different estrogen-responsive elements on the rat SR-B1 promoter and regulate its activity in response to estrogens (54). The negative regulators of SR-B1 include the nuclear receptor dorsal-sensitive sex adrenal hypoplasia congenital critical region on the X chromosome gene 1 (DAX-1), a protein that plays an important role in adrenal development (49), the Yin Yang 1 (YY1) transcription factor, which represses the activity of the SR-B1 promoter by inhibiting the binding of SREBP-1a (55), and the pregnane X receptor, which represses the human SR-B1 promoter activity in response to the pregnane X receptor agonists rifampicin and lithocholic acid (56). Most of the binding sites for these transcription factors reside within the 2.2 kb proximal 5′-flanking region of the SR-B1 promoter.

In steroidogenic tissues, SR-B1 expression was shown to be upregulated by ACTH (8, 44, 46, 57–59). As mentioned above, SF-1 was shown to mediate the regulation of SR-B1 gene expression through the cAMP–PKA pathway by binding to the promoter region of SR-B1. The SF-1 binding site in the human SR-B1 promoter (5′-CCAAGGCT-3′) resides 77 bp upstream of the transcription start site, and the SF-1 binding site (5′-TCAAGGCC-3′) in the rat SR-B1 promoter is located at −645 bp upstream of the translation start site. These two sites share 75% identity (48, 49). Both the human and rat SR-B1 promoters were shown to be active in mouse adrenocortical tumor Y1 cells, and mutagenesis analysis confirmed the involvement of SF-1 in regulating their promoter activity. The SF-1 binding motif in the rat SR-B1 promoter was shown to be involved in both basal and cAMP-induced regulation of SR-B1 gene expression. Further analysis of the functional domains in SF-1 revealed that both amino acids 448–461 and phosphorylation at Ser430 by PKA are involved in regulating the binding to the consensus sequence in the rat SR-B1 promoter. A recent report showed that when glucocorticoid levels are elevated, SR-B1 expression can also be inhibited by feedback regulation by glucocorticoid (60). In corticosterone-insufficient corticotrophin-releasing hormone knockout Crh (−/−) mice, there is an increase of SR-B1 mRNA levels in adrenal, and oral administration of corticosterone inhibited SR-B1 gene expression. Further studies reveal that the glucocorticoid receptor (GR) can suppress SR-B1 promoter activity. The region between −201 and −62 of the human SR-B1 promoter was shown to contain putative binding sites for transcriptional repressors that are involved in mediating glucocorticoid regulation of SR-B1 expression. However, examination into the mechanism of suppression suggested that GR suppression of SR-B1 in adrenal cells occurs through an indirect mechanism since no direct binding of GR to the SR-B1 promoter was observed. This was the first report showing that by suppressing SR-B1-mediated HDL cholesterol uptake, steroidogenic tissues maintain steroid hormone homeostasis when the endogenous levels of glucocorticoids are elevated.

Recently, in the search for the cellular and molecular mechanisms involved in the regulation of SR-B1 expression and function in steroidogenic cells, we demonstrated that two microRNAs, miRNA-125a and miRNA-455, can bind to specific sites in the 3′ UTR of SR-B1 mRNA and regulate the expression of SR-B1 (61). The expression of miRNA-125a and miRNA-455 is detected in steroidogenic tissue/cells, including adrenal, primary ovarian granulosa cells, and model Leydig cell lines. Both ACTH and cAMP downregulate the expression of miRNA-125a and miRNA-455. When either miRNA-125a or miRNA-455 is overexpressed or inhibited, the amount of SR-B1 protein expressed on the cell surface is decreased or increased, respectively, leading to SR-B1-mediated selective HDL uptake and SR-B1-supported steroid hormone synthesis being inhibited and stimulated, respectively. Therefore, our findings suggest that miRNA-125a and miRNA-455, in response to ACTH stimulation, act as SR-B1 attenuators to negatively regulate SR-B1 expression and SR-B1-mediated selective delivery of lipoprotein cholesterol in steroidogenic cells and, consequently, inhibition of SR-B1-supported steroidogenesis.

## Modulation of SR-B1 Protein Function by ACTH

SR-B1 facilitates HDL-CE selective uptake in two separate independent steps: binding of the lipid-rich lipoprotein to the extracellular domain (ECD) of SR-B1 and the delivery of the CEs from the hydrophobic core of the lipoprotein to the PM (62, 63). A specialized cell surface structure, termed “microvillar channels,” was reported to be induced by SR-B1 and shown to facilitate selective lipid transfer to inside the cell (64–67). Studies using electron microscopy demonstrated the presence of microvillar membrane domains in rat ovarian luteal, testicular Leydig, and adrenocortical cells. These domains form channels at the PM and various lipoproteins, including HDL, get trapped within the channels. Immunostaining using specific antibodies for SR-B1 revealed that SR-B1 is preferentially localized on these domains (65, 66). SR-B1 was also shown to be able to facilitate the formation of specific lipid rafts, hence change the properties of the PM and, in turn, affect the flux of free cholesterol (67). These lipid rafts were indicated to be necessary for the formation of the membrane microvillar channels, which are considered a trap for HDL particles for enhancing the efficiency of the selective uptake of HDL-CE. Interestingly, expression of SR-B1 and microvilli are under hormonal regulation; ACTH treatment increases, whereas dexamethasone treatment decreases, SR-B1 expression. Furthermore, the steady-state levels of adrenal microvilli are dictated by SR-B1. In response to SR-B1 deficiency, mouse adrenal microvilli become disorganized, and microvillar channels show a disrupted appearance along with substantially reduced binding of HDL particles to the cell surface (64–67).

In addition, experiments utilizing mutational analysis of SR-B1 or chimeras of CD36/SR-B1 have demonstrated that high affinity binding of lipoproteins to the ECD of SR-B1 is important, but not sufficient to mediate efficient lipid uptake (68). However, at the same time the ECD of SR-B1 does influence the efficient transfer of lipid by SR-B1 (69). This dichotomy is highlighted by the findings that some chemicals can increase lipoprotein binding to SR-B1 while actually blocking lipid transfer (70). Further studies show that the dominant characteristic of lipoprotein binding to SR-B1 involves protein–protein interactions between ligand and receptor. Many of the CE donors (HDL, apoA-I/phospholipid bilayer disks, and lipid-free apoA-I) for SR-B1 all share class A amphipathic helices that could be the structural feature to which SR-B1 is binding (68, 69). In addition, SR-B1 occurs as a multimeric complex with itself or other membrane proteins on the cell surface to facilitate lipid transfer, and the ECD of SR-B1 is essential for efficient CE transfer. ACTH has been shown to induce changes in the oligomeric status as well as protein interaction of SR-B1 and hence modulates SR-B1 protein function.

### ACTH Stimulation Modulates the Oligomerization of SR-B1

In response to ACTH stimulation, SR-B1 changes its oligomeric status to facilitate CE uptake (for simplicity, here, we use the term dimerization to include the multiple forms of the SR-B1 protein; i.e., dimers and higher order oligomers). In one of the earliest direct demonstrations of protein–protein interactions involving SR-B1, SR-B1 was shown to exist as homodimers in PMs isolated from rat adrenals stimulated with 17α-ethinyl estradiol (70). Subsequently, dimeric and higher order oligomeric forms of SR-B1 were shown to exist in all cells and tissues that display HDL-CE selective uptake activity (65, 71, 72). In normal rat adrenal tissue, SR-B1 exists primarily in a monomeric form with some dimer formation. Upon ACTH stimulation, there is a significant increase in the dimerization/oligomerization of SR-B1 along with increased selective CE uptake. On the other hand, dexamethasone-mediated suppression of ACTH leads to dramatic loss of SR-B1, SR-B1 dimers/oligomers, and HDL-CE selective uptake activity. When combined with the substantial architectural alterations of the cell surface as related to microvillar formation, these findings indicate that SR-B1 dimer/oligomer formation appears to have significant implications for the expression of the functional properties of SR-B1.

Studies in adrenal cells and other steroidogenic tissues have established a strong correlation between the levels of SR-B1 dimers and enhanced HDL-CE selective uptake activity. Co-immunoprecipitation with differentially epitope-tagged SR-B1s further confirmed that SR-B1 can exist as homodimers (71). In addition, in both native steroidogenic cell lines (endogenous) and in a heterologous insect cell overexpressing SR-B1, dimers/oligomers of SR-B1 were seen when cross-linking agents were added to the cell lysates (65). When cellular extracts from SR-B1 transfected HEK-293 cells or ACTH-treated Y1-BS1 cells were analyzed by size-exclusion chromatography and sucrose density centrifugation, a significant portion of SR-B1 eluted at peaks that correlate with the size of dimeric and oligomeric forms of SR-B1. Immunoelectron microscopy was used as an independent means for confirming the homodimerization of SR-B1. For these experiments, differentially epitope-tagged-SR-B1 proteins were co-expressed in HEK-293 cells, and the epitope-tagged proteins were subsequently immunostained and identified using two differently sized gold particles. The observed mixing and clustering of gold particles suggested that the proteins were localized to the same regions of the cell and that many of the gold particles were in extremely close proximity, i.e., within a distance for protein–protein interactions, as detected by fluorescent resonance energy transfer (FRET) technique. Similar results were obtained when Y1-BS1 mouse adrenocortical cells were transfected with differentially epitope-tagged-SR-B1 constructs. Interestingly, transfection of Y1-BS1 cells with SR-B1 in these experiments resulted in substantial architectural changes with the formation of microvillar structures. Gold-labeled secondary antibodies localized SR-B1 to these sites and revealed substantial dimer formation of this protein – shown by close contact between gold particles (71, 72).

Further investigations concentrated on the contribution of the cysteine residues in the ECD of SR-B1 either independently or in cooperation with the C-terminal domain on SR-B1 dimerization. SR-B1 contains a total of eight cysteine (C) residues (C21, C251, C280, C321, C323, C334, C384, and C470) and six of them are located in the ECD. Mutagenesis studies showed that C280, C321, C323, and C334 residues in the ECD are necessary for preserving normal SR-B1 (HDL) binding activity, selective CE uptake, and/or cell surface expression. Interestingly, mutation of any of these four cysteine residues to serine resulted in a robust induction of SR-B1 dimer formation, but, in contrast to normal SR-B1, these SR-B1 mutants lost their ability to mediate HDL-CE selective uptake. These results indicate that these cysteine residues are most likely essential for optimal HDL binding and selective CE uptake (73).

### ACTH Stimulation Regulates SR-B1 Interaction with Accessory Proteins

Adrenocorticotropic hormone treatment activates the cAMP–PKA signaling cascade, and we have recently shown that the expression of salt-inducible kinase 1 (SIK1), a serine/threonine kinase that belongs to the stress- and energy-sensing AMPK family of kinases, is also rapidly induced in Y1 adrenal cells in response to ACTH *via* the cAMP–PKA signaling cascade. Previously, it had been suggested that an increased level of SIK1 expression inhibits adrenal steroidogenesis by repressing the cAMP-dependent transcription of steroidogenic proteins, CYP11A1 and StAR, by attenuating CREB transcriptional activity (74). In contrast, we showed that SIK1 stimulates adrenal steroidogenesis by modulating the selective HDL-CE transport activity of SR-B1. Overexpression of SIK1 increases cAMP-stimulated and SR-B1-mediated selective HDL-BODIPY-CE uptake in cell lines without impacting SR-B1 protein levels, whereas knockdown of SIK1 attenuated cAMP-stimulated selective HDL-BODIPY-CE uptake. SIK1 forms a complex with SR-B1 by interacting with its cytoplasmic C-terminal domain, and *in vitro* kinase activity measurements indicate that SIK1 can phosphorylate the C-terminal domain of SR-B1. Among potential phosphorylation sites, SIK1-catalyzed phosphorylation of Ser496 is critical for SIK1 stimulation of the selective CE transport activity of SR-B1. Mutational studies further demonstrated that both the intact catalytic activity of SIK1 and its PKA-catalyzed phosphorylation are essential for SIK1 stimulation of SR-B1 activity. Finally, overexpression of SIK1 caused time-dependent increases in SR-B1-mediated and HDL-supported steroid production in Y1 cells; however, these effects were lost with knockdown of SR-B1. It should be noted that, as opposed to these stimulatory effects of SIK1 on SR-B1 function, we confirmed that SIK1 does suppress CREB activity, which could contribute to the inhibition of steroidogenesis under some conditions. Taken together, these studies establish a role for SIK1 in the positive regulation of selective HDL-CE transport function of SR-B1 and steroidogenesis and suggest a potential mechanism for SIK1 signaling in modulating SR-B1-mediated selective CE uptake and associated steroidogenesis.

Many studies have also indicated that accessory proteins are crucial for the proper cellular expression of SR-B1 and SR-B1-mediated HDL-CE transport as well as other functions (75–83). For example, it has been shown that PDZK1/NHERF3 regulates hepatic SR-B1 stability and steady-state protein levels. Interestingly, PDZK1/NHERF3 is neither expressed nor essential for SR-B1 abundance or its cellular localization in steroidogenic cells of the adrenal gland, ovary, and testis (77). Recently, we have shown that two other NHERF family members, NHERF1 and NHERF2, negatively regulate the expression and function of SR-B1 in steroidogenic cells of the adrenal and gonads in response to ACTH (84). Specifically, we showed that ACTH treatment decreases NHERF1 and NHERF2 protein levels in rat adrenals and increases SR-B1 function. Co-immunoprecipitation, colocalization, bimolecular fluorescence complementation, and mutational analysis all indicated that NHERF1 and NHERF2 form complexes with SR-B1 protein and, as a result, inhibit SR-B1-mediated selective CE transport and steroidogenesis. Moreover, we demonstrated that the structural components required for NHERF1/2 to interact with SR-B1 included an intact COOH-terminal PDZ recognition motif (EAKL) in SR-B1 as well as the PDZ1 or PDZ2 domain of NHERF1, the PDZ2 domain of NHERF2, or the MERM domains of NHERF1/2. The *de novo* synthesis of SR-B1 was also inhibited by both NHERF1 and NHERF2 (84). In contrast to NHERF1 and NHERF2, NHERF4 had no effect on selective HDL-CE uptake or steroidogenesis. Altogether, these experiments demonstrated that NHERF1 and NHERF2 bind SR-B1 and negatively regulate SR-B1 expression, selective CE transport, and steroidogenesis (84).

## ACTH and Regulation of SR-B1 Under Pathophysiological Conditions

Since ACTH tightly regulates SR-B1 gene transcription, it should be expected that any pathophysiological conditions that affect ACTH levels would also impact SR-B1 gene expression in an identical manner. However, there is the possibility that altered SR-B1 function may represent an adaptive response to cope with stressful conditions. Indeed, results from studies of mice that underwent chronic psychosocial stress exhibited an exaggerated adrenal corticosterone response along with elevated SR-B1 protein levels (85). On the other hand, since adrenal cholesterol uptake is required for the production of anti-inflammatory glucocorticoids, gene deletion of functional SR-B1 in adrenals results in impaired steroid synthesis (86). Other studies using SR-B1 knockout mice demonstrated that SR-B1 can protect mice against endotoxemia (87). There is an uncontrolled robust inflammatory cytokine response in the SR-B1 deficient animals, and they exhibit higher lethality when challenged with LPS-doses that induce endotoxic shock. Furthermore, these animals also exhibit a dysregulated adrenal glucocorticoid-mediated stress response to fasting. In addition, fasting-induced elevated levels of serum ACTH were the consequence of adrenal glucocorticoid insufficiency in SR-B1 knockout mice (86). Finally, when SR-B1 was only selectively deleted in adrenocortical cells in a tissue-specific manner, the animals had impaired rates of glucocorticoid secretion in response to stress, especially when they were subjected to an endotoxin challenge; these animals with SR-B1 ablated only in adrenocortical cells showed enhanced local and systematic inflammatory response, blunted activation of atrophy genes in skeletal muscle, and have a high incidence of mortality (88). In the setting of acute stress, where the release of corticosterone in rodents or cortisol in humans peaks rapidly and declines quickly, it is likely that posttranslational regulation of SR-B1, such as protein phosphorylation and dimerization, could be more important in the regulation of steroidogenesis than transcriptional control. In contrast, under chronic stress conditions, mechanisms regulating SR-B1 at the transcriptional level and/or through miRNAs would appear to be more relevant.

Some of the adrenal steroid hormones, i.e., glucocorticoids, display robust daily variations in circulation under the circadian control by ACTH, and their rhythmic activity is considered to play important roles in whole body health and disease (89, 90). Ablation of one of the genes involved in circadian control, BMAL1, results in loss of circadian regulation of glucocorticoids. The BMAL1-deficient animals showed impaired response to ACTH regulation of adrenal function and downregulation of genes involved in cholesterol transport, such as StAR and LDLR, in adrenals (89). There is a well-accepted concept of the adrenal clock, with evidence of differential responses between males and females (91). While studies have shown a circadian variation in the expression of several genes involved in regulating the steroid production, no publications have specifically examined whether SR-B1 expression and/or function displays a circadian rhythm, but this is likely in view of the known regulation of SR-B1 by ACTH.

**Figure 3:**
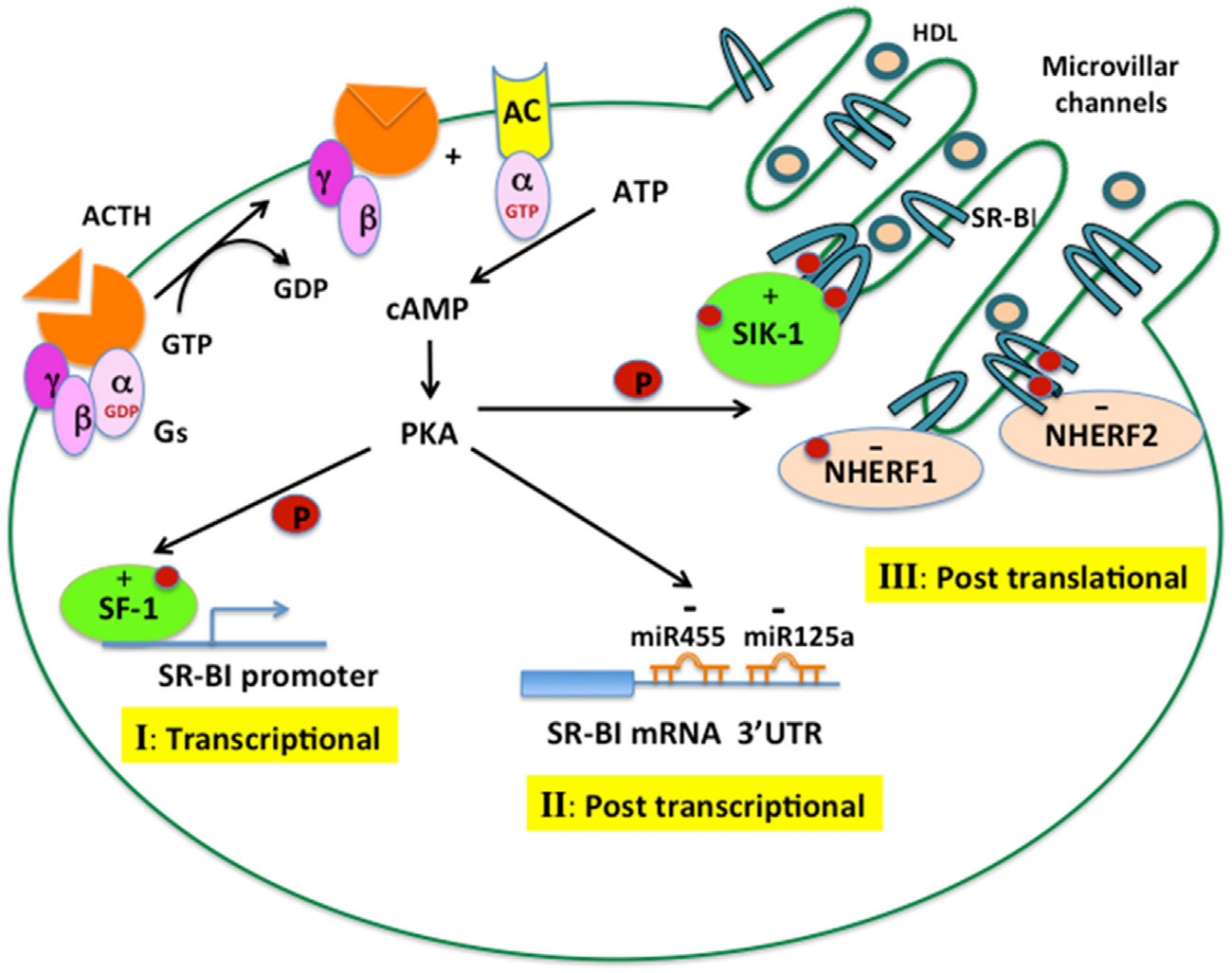
**ACTH regulation of SR-B1 in the adrenal**. ACTH binds to its G protein-coupled receptor, leading to the activation of adenylate cyclase, which generates cAMP and activates cAMP-dependent protein kinase (PKA). The cAMP–PKA signaling cascade can regulate SR-B1 expression and function at different levels. (I) Transcriptional control: PKA increases the phosphorylation of transcription factors, such as SF1, leading to increased promoter activity of SR-B1. (II) Posttranscriptional control: PKA increases the expression of miRNA-125a and miRNA-455, which can bind to the 3′ UTR of SR-B1 mRNA and negatively regulate SR-B1 expression. (III) Posttranslational control: PKA induces the oligomerization of SR-B1 and stimulates an interaction with SIK1, leading to phosphorylation of SR-B1, both events resulting in increased SR-B1 protein function. PKA can also increase the interaction of SR-B1 with NHERF1 and NHERF2, which negatively regulate SR-B1 function.

In summary, through binding to its G protein-coupled receptors, leading to the activation of adenylate cyclase, which generates cAMP and activates PKA, ACTH exerts tight regulation of SR-B1 function in the adrenal at three different levels. As illustrated in Figure 3, increased cAMP–PKA signaling can increase the phosphorylation of transcription factors, such as SF1, and stimulate promoter activity of SR-B1. ACTH can increase the expression of miRNA-125 and miRNA-455, which can bind to the 3′ UTR of SR-B1 and result in suppressed expression and function of SR-B1 protein. Increased cAMP–PKA signaling can induce oligomerization of SR-B1 as well as stimulate phosphorylation of SIK1, which, in turn, increases the phosphorylation of SR-B1 and results in increased function of SR-B1 protein. Meanwhile, ACTH can also induce the expression of NHERF1 and NHERF2, both of which can bind to SR-B1 and negatively regulate SR-B1 function. Altered SR-B1 function may represent an adaptive response to cope with stressful conditions, as demonstrated by ablation of SR-B1 resulting in disturbed anti-inflammatory glucocorticoid homeostasis and impaired steroid synthesis.

## Author Contributions

All authors listed, have made substantial, direct and intellectual contribution to the work, and approved it for publication.

## Conflict of Interest Statement

The authors declare that the research was conducted in the absence of any commercial or financial relationships that could be construed as a potential conflict of interest.
